# P21 A Phase 3 randomized, double-blind, active comparator-controlled study to evaluate the safety, tolerability and immunogenicity of a 21-valent PCV, V116, in pneumococcal vaccine-naive adults (STRIDE-3)

**DOI:** 10.1093/jacamr/dlad143.025

**Published:** 2024-01-03

**Authors:** Christopher Bruno, Erik Buntinx, Enrique Pelayo, Jackie M Kamerbeek, Diego Garcia-Huidobro, Elizabeth A Barranco-Santana, Folke Sjoberg, Joon Young Song, David Greenberg, Carlos G Grijalva, Walter A Orenstein, Leslie Morgan, Doreen Fernsler, Weifeng Xu, Muhammad Waleed, Jianing Li, Ulrike K Buchwald, Heather L Platt

**Affiliations:** Merck & Co. Inc., Rahway, NJ, USA; Anima Research Center, Alken, Belgium; Amri, Miami, FL, USA; P3 Research Ltd, Tauranga, New Zealand; Pontificia Universidad Catolica de Chile, Santiago, Chile; Ponce Health Sciences University, Ponce, Puerto Rico; Karolinska University Hospital, Stockholm, Sweden; Korea University College of Medicine, Seoul, Korea; Soroka University Medical Center, Beer-Sheva, Israel; Vanderbilt University Medical Center, Nashville, TN, USA; Emory University, Atlanta, GA, USA; Merck & Co. Inc., Rahway, NJ, USA; Merck & Co. Inc., Rahway, NJ, USA; Merck & Co. Inc., Rahway, NJ, USA; Merck & Co. Inc., Rahway, NJ, USA; Merck & Co. Inc., Rahway, NJ, USA; Merck & Co. Inc., Rahway, NJ, USA; Merck & Co. Inc., Rahway, NJ, USA

## Abstract

**Background:**

Pneumococcal disease (PD) prevention remains an unmet medical need in adults. V116 is an investigational pneumococcal conjugate vaccine (PCV) containing the most prevalent serotypes associated with PD in adults in regions with established paediatric vaccination programmes. This Phase 3 study evaluated safety, tolerability, and immunogenicity of V116 compared with PCV20 in adults.

**Methods:**

Pneumococcal vaccine-naive adults ≥18 years were eligible. Cohort 1 (≥50 years, *n*=2362) was stratified by age (50–64, 65–74, 75–84 and ≥85) and randomized 1:1 to receive 1 dose of V116 or PCV20, and cohort 2 (18–49 years, *n*=301) was randomized 2:1 to receive 1 dose of V116 or PCV20. Pneumococcal serotype-specific opsonophagocytic activity (OPA) and IgG responses were measured at baseline (Day 1) and 30 days post vaccination (Day 30). Primary objectives included assessment of (i) non-inferiority of immune responses for the serotypes common to V116 and PCV20 in cohort 1; (ii) superiority of serotypes unique to V116 compared with PCV20 in cohort 1; and (iii) immunobridging from adults 18–49 to adults 50–64 for all 21 serotypes in V116. Safety was evaluated by the proportion of participants with adverse events (AEs).

**Results:**

V116 is non-inferior to PCV20 for the 10 serotypes common to both vaccines as measured by serotype-specific OPA geometric mean titres (GMTs) at Day 30. V116 is superior to PCV20 for 10 of 11 unique serotypes as measured by OPA GMTs at Day 30 as well as based on the proportions of participants with a ≥4-fold rise in OPA from Day 1 to Day 30. V116 elicits comparable immune responses in participants 18–49 years compared with participants 50–64 years for the 21 serotypes in V116 as assessed by serotype-specific OPA GMTs at Day 30, successfully demonstrating immunobridging. In total, 61.7% and 67.2% of participants vaccinated with V116 and PCV20, respectively, had ≥1 AE. There were no vaccine-related serious AEs or vaccine-related deaths.

**Conclusions:**

V116 elicits immune responses that are non-inferior to PCV20 for the common serotypes and superior to PCV20 for 10 of 11 unique serotypes in V116 and has a safety profile comparable to PCV20. This pivotal study supports V116 as a novel population-specific vaccine for the prevention of PD in adults.

